# The Concept of Drug-Resistant Epileptogenic Zone

**DOI:** 10.3389/fneur.2019.00558

**Published:** 2019-05-31

**Authors:** Chunbo Zhang, Patrick Kwan

**Affiliations:** ^1^School of Pharmacy, Nanchang University, Nanchang, China; ^2^Department of Neuroscience, Alfred Hospital, Monash University, Melbourne, VIC, Australia; ^3^Departments of Medicine and Neurology, Royal Melbourne Hospital, The University of Melbourne, Parkville, VIC, Australia

**Keywords:** epilepsy, epileptogenic zone, epilepsy surgery, drug resistance, seizure outcome, drug withdrawal

## Abstract

Resective surgery is the most effective way to treat drug-resistant epilepsy. Despite extensive pre-surgical evaluation, only 30–70% patients would become seizure-free after surgery. New approaches and strategies are needed to improve the outcome of epilepsy surgery. It is commonly observed in clinical practice that antiepileptic drugs (AEDs) could maintain seizure freedom in a large proportion of patients after surgery, who were uncontrolled before the operation. In some patients cessation of AEDs leads to seizure recurrence which, in most cases, can be controlled by resuming AEDs. These observations suggest that the surgery has converted the epilepsy from drug-resistant to drug-responsive, implying that the operation has removed the brain tissue accounting for pharmacoresistance, rather than the pathological substrate of epilepsy (at least not completely). Based on these observations, it is hypothesized that there is a drug-resistant epileptogenic zone (DREZ) which overlaps with the epileptogenic zone (EZ), and has both epileptogenic and drug-resistant properties. DREZ is necessary and sufficient to cause drug-resistant epilepsy, and its remove would render the epilepsy drug-responsive. Testing the hypothesis requires the development of new methods to define the DREZ, which may be used to guide surgical planning when the epileptogenic zone cannot be completely excised. This concept can also help understand the mechanisms of drug-resistant epilepsy, leading to new therapeutic strategies.

## Introduction

Affecting ~68 million people worldwide, epilepsy is one of the most common chronic neurological disorders. Up to one-third of epilepsy patients do not respond to antiepileptic drugs (AEDs) (1). In selected patients, surgery is the most effective therapeutic modality, leading to seizure freedom and associated improvement in quality of life (2). Based on the widely accepted concept that seizures originate from the epileptogenic zone (EZ), resective surgery aims to remove this zone as complete as possible (3). In doing so, the epilepsy would be “cured,” and the patient would no longer require drug treatment.

Despite the advances in diagnostic and surgical techniques, 30–40% patients continue to have seizures after resective surgery (4). The reasons for the unfavorable outcome of surgery are complex and may include incomplete resection of the EZ, as well as re-kindling of the epileptogenic circuit, incomplete interruption of the complex network, and multiple, widespread foci or aberrant neuronal network (5). The latter considerations reflect the rising appreciation of epilepsy as a network (6).

Importantly, in patients who have become seizure-free post-surgery, only around 45% of them can successfully withdraw AED treatment, while many remain on medications to maintain seizure freedom. This is in contrast to the situation before surgery when they had seizures despite taking multiple AEDs. In many such patients, cessation of AEDs would lead to seizure recurrence and resuming AEDs would regain seizure control (7). In other words, instead of being a “curative” treatment, the operation has converted the epilepsy from drug-resistant to drug-responsive. This implies that the surgery may have removed the pathological tissue accounting for pharmacoresistance, rather than the EZ *per se* (at least not completely). Moreover, incomplete resection of pathological tissue or epileptiform discharging zone could still lead to seizure freedom in some cases (8). Accordingly, preoperative neuroimaging, histopathology of resected brain tissue, and epileptic animal models showed that EZ is not always homogeneity (3, 9, 10).

Based on these observations, it is hypothesized that there is a drug-resistant epileptogenic zone (DREZ) in the brains of patients with drug-resistant epilepsy. The DREZ forms part of the EZ and has both drug-resistant and epileptogenic characters. The DREZ is necessary and sufficient to cause drug-resistant epilepsy, and its removal can render the drug-resistant epilepsy drug-responsive.

This article aims not to discuss the definition of EZ, which continues to evolve (6), but to expound the concept of the DREZ by reviewing the seizure outcome after resective epilepsy surgery, summarizing the findings supporting the presence of DREZ, and discussing how it might be identified. If demonstrated to be correct, this hypothesis could have practical implications for presurgical evaluation and the diagnosis of drug-resistant pathological tissues, which may lead to improved epilepsy surgery outcome. This concept would also facilitate tailoring the resection of the DREZ, particularly in patients whose EZ cannot be completely excised due to overlapping with the eloquent cortex, multifocal EZ, and widespread EZ.

## Outcome of Resective Epilepsy Surgery

### Seizure Freedom After Resection

Resective surgery is considered a treatment option for patients with drug-resistant focal epilepsy. Randomized controlled trials have demonstrated that, in appropriately selected patients, surgery is superior to medical therapy alone (11–13). The rates of seizure freedom after surgery have been reported in a large number of case series, clinical trials and systematic reviews. Overall, 55–70% of patients who had temporal lobe resection and 30–50% of patients who underwent extratemporal resection, would become seizure-free (4). A landmark randomized controlled trial reported that 58% of patients (over 16 years old) who had temporal lobe resection were seizure-free at 1-year follow-up, compared to 8% in the medical group (11). This was confirmed in a subsequent randomized controlled trial which reported that 77% of pediatric epilepsy patients were seizure free at 1 year follow up after surgery, compared to 7% in the medical group (13). A meta-analysis included 16,253 patients who had epilepsy surgery from 177 studies (8). The overall proportion of patients with good outcome (>1-year seizure control at Engel class 1 or seizure-free status) was 65% (10,518 in 16,253 patients), ranging from 13.5 to 92.5% in different centers (8). Potential reasons for the variation might include differences in preoperative evaluation protocols, types of epilepsy and operation, intraoperative decision, post-operative medication and classification of seizure outcome.

### Drug Withdrawal After Surgery

Medication usage is an important parameter when evaluating the outcome of epilepsy surgery. Most patients continue to take AEDs to avoid seizure recurrence at the first 6–24 months after surgery (8), after which some may elect to reduce or withdraw treatment. A retrospective analysis of 202 patients found that patients took fewer AEDs after compared to before surgery (14). In an analysis from 15 centers of 766 children who attempted drug withdrawal after becoming seizure-free post-surgery, 411 (54%) were able to completely discontinue AEDs at the last follow-up. Of the 87 patients who experienced seizure recurrence and retook AEDs, 61 (70%) regained seizure control (7).

Table 1 summarizes the studies on seizure outcome in patients attempted drug withdrawal after surgery. Around 10–50% of patients had seizure recurrence during and after drug withdrawal. Early drug withdrawal was associated with an increased risk of seizure recurrence (7). After resuming AEDs, around 40–90% of patients regained seizure freedom. These results imply that in these patients, the operation has removed the pathological tissue responsible for pharmacoresistance, while the remaining epileptogenic zone is drug-responsive. However, drug withdrawal is not a standard procedure after epilepsy surgery (15, 29). A large proportion of patients did not attempt drug withdrawal (7, 15, 30). A randomized clinic trial is ongoing, and no data is published based on the randomized clinic trial so far (29). The statistical data summarized here may not represent the actual seizure outcome after drug withdrawal (15, 30). Further studies are needed to investigate the actual seizure outcome in patients attempted drug withdrawal after surgery.

**Table 1:** Outcome of drug withdrawal after successful epilepsy surgery.

**Study**	**Surgery cases in the study**	**Patients attempting drug withdrawal**	**Study design** **age (years);** **classification of surgery outcome;** **diagnostic evaluation; surgery types;** **selection criteria of patients attempting drug withdrawal**	**Duration of post-operative follow-up (years)**	**Seizure recurrence during and after drug withdrawal**	**Seizure-free and AED-free patients at last follow-up**	**Patients re-start AED after seizure recurrence**	**Seizure free after re-starting AEDs**
Rathore et al. (15)	384	326	12 (7–17) seizure-free outcome corresponded to a seizure score of 0 to 1, equivalent to Engel class IA and ILAE class 1 EEG, MRI ATL over 10-year MTLE, ≥5-year follow-up	11.9 ± 2.8 (7–17)	92 (28.2%, 92/326)	207 (63.5%, 207/326)	92	79 (85.7%, 79/92)
Yardi et al. (16)	609	380	86% adult Engel class MRI, PET, EEG temporal lobectomy, AHE, tailored cortical resection NA	0.5–16.7	202 (53%, 202/380)^*^	68 (18%, 68/380)^*^	NA	NA
Boshuisen et al. (7)	766	766	<18 years old Engel class, ILAE class MRI, EEG lobar resection, hemispherectomy, multilobar resection NA	5.1 ± 2.5	95 (12%, 95/776)	344 (45%, 344/776)	87^#^	61 (70%, 61/87)
Menon et al. (17)	106	94	7.4 ± 5.5 (0.6–27) NA MRI, EEG, ECoG lesionectomy, lobectomy ≥2 years of post-operative follow-up, seizure-free patients or early post-operative recurrence subsequently became seizure-free ≥2 years	4.6 ± 2.2 (2–11)	44 (46.8%, 44/94)	26 (27.7%, 26/94)	44	30 (68%, 30/44)
Pimentel et al. (18)	67	38	43.6 ± 14.02 Engel Class NA temporal lobe resection AHE with HS, Engel class IA ≥1 year after surgery	4.8 ± 2.9 (1.1–13.2)	12 (31.6%, 12/38)	NA	12	10 (83.3%, 10/12)
Rathore et al, (19)	310	258	27.1 ± 9.2 Engel class, ILAE Class EEG, MRI ATL seizure-free or patients without any consciousness impairing seizures ≥2 years.	8 ± 2	64 (24.8%, 64/258)	163 (63.2%, 163/258)	64	56 (87.5%, 56/64)
Maehara et al. (20)	40	17^*^	30.0 ± 11.9 (9–59) Engel Class MRI, PET, EEG. TLE seizure-free ≥1 year after surgery	6.8 (3.6–10.4)	3 (17.6%, 3/17)	14 (82%, 14/17)	3	3 (100%, 3/3)
Park et al. (21)	223	147	28.2 ± 9.6 (*n* = 78, relapse), 25.6 ± 10.6 (*n* = 69, no relapse) NA MRI, EEG, PET, SPECT lesionectomy, lobectomy NA	7 (2–12.6)	78 (53.1%, 78/147)	59 (40%, 59/147)	75	36 (48%, 36/75)
Kerling et al. (22)	34	34	35.6 ± 10.8 (20–62) Engel class, ILAE class MRI, EEG NA completely seizure free without any auras ≥1 year after surgery	5	8 (23.5%, 8/34)	17 (50%, 17/34)	8	5 (62.5%, 5/8)
Boshuisen et al. (23)	109	84	8.76 ± 5.1 NA NA hemispherectomy, temporal resection, extratemporal resection NA	8 (3–17)	5 (6%, 5/84)	65 (77.4%, 65/84)	5	2 (40%, 2/5)
Lachhwani et al. (24)	97	68	11 (0.25–18) NA NA temporal, frontal or frontoparietal, multilobar involving temporal, parietal and occipital cortex, hemispheric seizure free >6 months, and ≥1 year follow-up after discontinuation of AEDs	NA	11 (16.2%, 11/68)	57 (83.8%, 57/68)	11	7 (63.6%, 7/11)
Berg et al. (25)	396	129	36 remission: ≥1 year seizure free with or without auras NA neocortical, medial-temporal ≥1 year seizure free with or without auras	NA	41 (31.8%, 41/129)	43 (33.3%, 43/129)	37	26 (70%, 26/37)
Kim et al. (26)	88	60	27 (11–41) success: complete seizure free without aura NA temporal lobectomy refractory TLE, ≥3 years follow-up after surgery, complete resection of the lesions	6.5 (3–12)	20 (33%, 20/60)	37 (62%, 37/60)	20	9 (45%, 9/20)
Griffin et al. (27)	30	22	30 (10–56) Engel Class MRI ATL NA	3.4 ± 2.7	6 (27.3%, 6/22)	2 (9%, 2/22)^*^	6	3 (50%, 3/6)
Schiller et al. (28)	210	180	31.9 (9–55) NA MRI, EEG, ECoG temporal lobe resection, extratemporal resection seizure and aura-free for ≥1 year after surgery	5.1 ± 0.3 (3–18)	35 (19.4%, 35/180)	62 (34.4%, 62/180)	22^*^	20 (91%, 20/22)^*^

* *data was not completely shown*.

# *unknow if restarted AEDs in some seizure recurrence patients*.

*NA, not available*.

*ATL, anterior temporal lobectomy; AHE, selective amygdalohippocampectomies; HS, hippocampal sclerosis*.

Some patients with seizure recurrence during or after drug withdrawal did not regain seizure freedom after retaking AEDs (Table 1). Several reasons might be involved. First, seizure recurrence may change microenvironment in the brain, such as upregulation of multiple drug transporters and alteration of drug targets, leading to the generation of new drug-resistant zones and uncontrolled seizures (31–35). Second, the recurrence of refractory epilepsy may be caused by incomplete removal of the potential epileptogenic zone (5, 36, 37), which will result in epilepsy recurrence even without drug withdraw. The third possible explanation is the development of a new epileptogenic zone, which is induced by multiple factors, such as brain injury and infection during or after surgery, the accumulation of tendency of excitatory connections, the change of subcellular structure of pathological tissue, genetics, and so on (5, 38, 39). However, the mechanisms underlying this phenomenon is unclear, and more investigations are needed.

### Incomplete Resection

Incomplete resection of the epileptogenic zone is one of the most common reasons for surgical failure (40). The definition of completeness varies across studies, depending on post-operative MRI diagnosis, use of intracranial electrodes, or the type of surgery performed (8, 41, 42). Complete resection of tissue with epileptiform discharges is usually associated with good seizure outcome (43). A meta-analysis showed that 74% (1,277/1,716) patients with complete resection achieved good outcomes, compared to 56% (725/1,297) with incomplete resection (8). Among 28 factors analyzed in 149 patients with focal cortical dysplasia, the completeness of resection of the epileptogenic zone was the only significant predictor for good surgical outcome (44).

Conversely, it is notable that a proportion of patients with incomplete resection of the pathological lesion could still become seizure-free. The rate of good outcome after incomplete resection varies from 20 to 80% across studies (Table 2) (8, 41, 44, 57, 58). The mechanism of this phenomenon is unclear. It is possible that the epileptogenic zone only occupied a part of pathological lesions, and removal of this area achieved seizure freedom. Another possibility is that the epileptogenic zone is not homogeneous (3). In the seizure-free patients with incomplete resection, the resected brain tissue was the part of the epileptogenic zone responsible for drug resistance (59–61). Removal of this area was, therefore, sufficient to render the epilepsy drug-responsive. However, the remaining lesion may still produce seizures in the absence of AEDs. The second phenomenon might explain why seizure recurs after drug withdrawal in the seizure-free patients.

**Table 2:** Seizure outcome according to the completeness of resection.

**Study**	**Total patients in study**	**Complete resection (No.)**	**Seizure free patients in complete resection %**	**Incomplete resection (No.)**	**Seizure free patients in incomplete resection %**	**Definition of completenesscriteria of completeness** **diagnostic methods**
Fujiwara et al. (45)	41	22	82% (18/22)	19	21% (4/19)	complete resection of epileptogenic high-frequency oscillations Intracranial EEG
Lee et al. (46)	40	32	100% (32/32)	8	62.5% (5/8)	complete resection of the lesion MRI
Kim et al. (47)	166	111	70% (77/111)	55	31% (17/55)	resection of all of ictal onset, persistent pathologic delta slowing, >1/s frequent spikes, intermittent gamma wave by intracranial EEG, MRI-visible lesion MRI, PET, EEG
Paolicchi et al. (48)	75	49	76% (37/49)	26	27% (7/26)	resection of all of EEG abnormalities and the structural lesion MRI, EEG
Kim et al. (49)	40	24	50% (12/24)	16	12.5% (2/16)	resection of all of ictal onset, persistent pathologic delta slowing, >1/s frequent spikes, intermittent gamma wave by intracranial EEG, MRI-visible lesion MRI, PET, EEG, SPECT
Jennum et al. (50)	64	50	70% (35/50)	14	43% (6/14)	resection of all of ictal focus MRI, EEG
Awad et al. (51)	47	18	94% (17/18)	29	34% (10/29)	“spike active” fully resected subdural EEG
Wyler et al. (52)	70	36	69% (25/36)	34	38% (13/34)	total hippocampectomy NA
Widdess-Walsh et al. (42)	48^*^	22	68% (15/22)	19	26% (5/19)	complete resection of ictal onset zone subdural EEG
Cossu et al. (53)	165	115	51% (59/115)	50	28% (14/50)	complete lesion removal SEEG, MRI
Jayakar et al. (54)	102^*^	63	56% (35/63)	38	24% (9/38)	removal of all regions of significant EEG and ictal SPECT abnormalities EEG, SPECT
Kanner et al. (55)	100^*^	25	52% (13/25)	74	40% (29/74)	extent of resection of mesial structures EEG, MRI, PET
Kloss et al. (56)	68^*^	26	81% (21/26)	30	17% (5/30)	complete removal of the MRI lesion MRI, PET, SPECT, EEG
O'Brien et al. (41)	36^*^	4	100% (4/4)	11	55% (6/11)	completely resection of SISCOM focus SPECT, EEG, MRI

*NA, not available*.

* *The completeness of surgery was unclassified or missed in some patients, or seizure outcome was missed follow-up in some cases*.

*SISCOM: subtraction ictal SPECT coregistered with MRI*.

### Hypothesis for the Drug-Resistant Epileptogenic Zone

**Figure 1:**
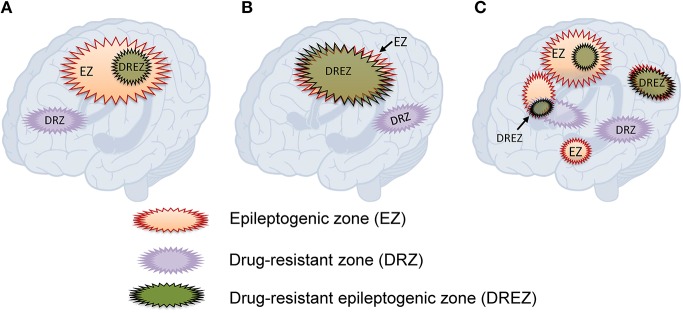
Hypothesized relationships between the drug-resistant epileptogenic zone and epileptogenic zone. **(A)** The drug-resistant epileptogenic zone is part of the epileptogenic zone. **(B)** The drug-resistant epileptogenic zone overlaps with the whole epileptogenic zone. **(C)** In multifocal epilepsy, the drug-resistant epileptogenic zone overlaps with some of the epileptogenic foci. Drug-resistant zone and epileptogenic zone may exclude from each other.

Based on the observations that: (1) resective surgery renders a large proportion of patients who were previously drug-resistant seizure-free, but that cessation of AEDs leads to seizure recurrence, and retaking AEDs can regain seizure control; and (2) a substantial proportion of patients with incomplete resection of the epileptogenic zone could achieve good outcome, we hypothesize that there is an area, which we term the “drug-resistant epileptogenic zone” (DREZ), responsible for drug-resistant ictogenesis in the brain (Figure 1). The DREZ may be defined as:

“the area that is necessary and sufficient to cause drug-resistant epilepsy, such that after its removal, the epilepsy will become controlled by antiepileptic drugs. This area may be located in one or several foci of the epileptogenic zone, and may be equal to or smaller than the epileptogenic zone.”

The definition of DREZ contains two criteria. First, the location and the function. DREZ should locate within the EZ, and the size of DREZ should be equal to or smaller than EZ. Second, DREZ should have both epileptogenic and drug-resistant properties. The epileptogenic function of DREZ is the same as the EZ, including initiation of seizures. Besides the epileptogenic function, DREZ should have drug-resistant character, such that AEDs are unable to control seizure onset in the DREZ. It is also hypothesized that some brain tissue only having drug-resistant property might extend beyond the EZ but is connected to the EZ as part of an excitatory network. In this case, the drug-resistant pathological tissue without epileptogenic property would not cause symptoms but would affect the circuit through its excitatory connections to the EZ. The concept of DREZ is based on current experiences and assumes that EZ and DREZ are static. The actual EZ and DREZ may dynamically change over time, as well as their connection in epilepsy networks.

### Potential Strategies to Identify DREZ

Based on the above consideration, two approaches could be employed to demonstrate the DREZ, with one to detect the epileptogenic zone and the other to identify the drug-resistant focus. In clinical practice, EEG recording, either by scalp or intracranial electrodes, is routinely combined with imaging modalities to define the epileptogenic boundary of DREZ (3). Identification of the drug-resistant focus requires an understanding of the mechanisms of pharmacoresistance, which remain unknown. There are several commonly proposed hypotheses of drug-resistant epilepsy (32), which have been summarized as the “drug transporter hypothesis,” “altered target hypothesis,” and “missing target hypothesis.” The drug transporter hypothesis is based on the observation of increased expression of efflux drug transporters (P-glycoprotein, BCRP, MRPs, and other ABC transporters) at the apical surface of cerebral capillary endothelial cells at the epileptogenic lesion. These transmembrane proteins are capable of transporting AEDs back into the capillary lumen, thereby reducing drug concentration and efficacy at the pathological area. The “altered target hypothesis” is based on the finding of altered neuronal molecular targets of AEDs, leading to reduced sensitivity to AEDs. Lastly, the “missing target theory” hypothesizes that the AEDs do not target the real pathogenic processes, such as autoimmune inflammation (32).

Among these hypotheses, the transporter hypothesis is arguably the most intensely investigated. While there has been much debate on the merit of overcoming pharmacoresistance by inhibiting their functions, these drug transporters have been consistently shown to be over-expressed in a range of epileptogenic pathologies. Among these transporters, P-glycoprotein is the most widely studied. Tishler et al. was the first group to report increased mRNA of *ABCB1*, which encodes P-glycoprotein, in the brain tissues resected from patients with drug-resistant epilepsy (62). Our group demonstrated that the overexpression of P-glycoprotein in the resected tissues was associated with seizure recurrence after epilepsy surgery (63). *In vivo* evidence also showed overactivity of P-glycoprotein in blood-brain barrier of refractory epilepsy patients (34). A PET study showed reduced brain uptake of (R)-[^11^C]-verapamil, a substrate of P-glycoprotein, in drug-resistant epilepsy patients, compared with seizure-free patients (34), implying increased activity of the drug transporter. A wide range of AEDs have been reported to be substrates of P-glycoprotein (64).

Collectively, these observations suggest that the overexpression of P-glycoprotein in the epileptogenic zone might be used as a candidate biomarker to identify the DREZ, and that the identification of brain tissues having both P-glycoprotein overexpression and seizure-onset characters could be a viable strategy to verify the existence of DREZ. If confirmed, identification of the DREZ may be used to guide surgical resection, particularly when the epileptogenic zone cannot be completely resected, for instance due to overlapping with eloquent cortex, multifocal EZ, or widespread EZ. To achieve this goal, methods to detect the overexpression of transporters intraoperatively would need to be developed, for instance by using fluorescent substrates of the transporters and dye-labeled monoclonal antibody (65, 66).

### The Correlation Between DREZ and Other Concepts

The concept of the epileptogenic zone continuously evolves based on knowledge, technics, and adaptability (6). In the 1950s, Wilder and Penfield defined the concept of epileptogenic lesion (67). Rasmussen et al. expended and proposed the tertiary localization concept (68, 69). In the 1960s, Talairach and Bancaud proposed “epileptogenic zone” to represent “the site of the beginning of the epileptic seizures and of their primary organization” (70). In 1993, Luders et al. defined the “epileptogenic zone” as “the area of cortex that is necessary and sufficient for initiating seizures and whose removal (or disconnection) is necessary for complete abolition of seizures” (3). In the 2000s, the concept of “epilepsy networks” became more acceptable. Spencer defined it as “A network [is] a functionally and anatomically connected, bilaterally represented, set of cortical and subcortical brain structures and regions in which activity in any one part affects activity in all the others” (71). These concepts are meaningful and have been used as the principle to guide epilepsy surgery. However, these concepts still have some limitations in guiding the ideal resection of pathological tissues. First, the margin of different theoretical zones is difficult to measure, such as tertiary localization, the ictal onset zone, the irritative zone, and potential epileptogenic zone. Second, theoretically EZ cannot be directly measured. Presurgical evaluation and intraoperative measurement can only delineate the potential pathological tissue. Surgeons usually try to extend the resection area in order to remove the entire epileptogenic zone based on their experience, which may increase the complications. The concept of DREZ tries to import the parameter of drug-resistance to help precisely tailor the resection area, especially in patients whose EZ cannot be completely excised due to overlapping with the eloquent cortex, multifocal EZ, and widespread EZ. It may also suggest the different medical treatment for patients with complete removal of EZ or complete removal of DREZ but still has some EZ left. The DREZ concept may also facilitate the discovery of new biomarkers in identifying the essential resective area.

However, there are limitations to the concept of DREZ. First, the mechanisms underlying drug-resistant epilepsy are still unclear. Although multiple hypotheses are involved, we still lack substantial evidence to verify them. Second, the drug-resistant zone may be dynamic in time and space. Multiple factors may trigger the generation of a new drug-resistant zone. The relative position between drug-resistant zone and EZ may dynamically change over time. Third, the methods to detect the drug-resistant zone are limited. Further studies are needed to investigate the relationship between DREZ, EZ, and epilepsy networks, as well as their applications in facilitating the success rates of epilepsy surgery.

### Other Possibilities for Post-Surgical Seizure Recurrence

The mechanisms underlying the recurrence of seizures after surgery are complex. Besides the incomplete resection of DREZ, other possibilities include re-kindling of the epileptogenic circuit, incomplete interruption of the epileptogenic circuit, a new generation of drug-resistant pathological zone (2, 5). During or after surgery, there may be re-kindling phenomenon over time in the remaining epileptogenic network, leading to seizure recurrence. New drug-resistant zone may also be induced during surgery or post-surgery. Multiple parameters may be involved in the generation of new drug-resistant zone, such as surgical trauma, inflammation, infection, excitatory neurotransmitters (32, 72, 73). Other factors involved in the abnormal circuit may also affect the prognosis of epilepsy surgery, such as the residual structural lesions, local drug pharmacokinetics and pharmacodynamics, or other pathological tissues (32, 36, 37, 40). The “running down phenomenon” suggests another possibility that surgery reduced the seizure tendency, which decreases the autonomous stimulation of seizures (74, 75). In this concept, the epileptogenic zone was not entirely removed in some drug-responsive patients, but the remaining epileptogenic tissue was not large enough to trigger seizures, suggesting the existence of a low threshold epileptogenic area. This area might be part or functionally correlated with DREZ. More studies are needed to investigate the mechanisms of recurrence of seizures post-surgery.

## Conclusion

Epilepsy surgery is the most effective procedure to treat patients with pharmacoresistant epilepsy. A large proportion of drug-resistant patients could become drug-responsive after surgery. Withdrawal of AEDs is associated with seizure recurrence, and resumption of AEDs could regain seizure control. While the mechanisms for seizure recurrence post-surgery are complex, we propose that there is a zone having both drug-resistant and epileptogenic characters in the brain of drug-resistant epilepsy patients. By removing this area, drug-resistant epilepsy would become drug responsive.

If correct, this hypothesis would fundamentally alter our approach to pre-surgical evaluation. Instead of aiming to delineate the complete epileptogenic zone, investigations would include defining the DREZ as the minimal tissues to remove to render the epilepsy drug-responsive. This approach may improve the outcome of epilepsy surgery, especially in patients whose EZ cannot be completely excised. It can also help understand the mechanisms of drug-resistant epilepsy, and develop new treatment strategies.

## Data Availability

All datasets analyzed for this study are included in the manuscript and the supplementary files.

## Author Contributions

CZ: design and conceptualized study, analyzed the data, draft the manuscript for the intellectual content. PK: design and conceptualized study, revised the manuscript for intellectual content.

### Conflict of Interest Statement

The authors declare that the research was conducted in the absence of any commercial or financial relationships that could be construed as a potential conflict of interest.
